# Hybrid FPGA–CPU-Based Architecture for Object Recognition in Visual Servoing of Arm Prosthesis

**DOI:** 10.3390/jimaging8020044

**Published:** 2022-02-12

**Authors:** Attila Fejér, Zoltán Nagy, Jenny Benois-Pineau, Péter Szolgay, Aymar de Rugy, Jean-Philippe Domenger

**Affiliations:** 1 Laboratoire Bordelais de Recherche en Informatique, University of Bordeaux, CEDEX, 33405 Talence, France; jenny.benois-pineau@u-bordeaux.fr (J.B.-P.); jean-philippe.domenger@labri.fr (J.-P.D.); 2 Faculty of Information Technology and Bionics, Pázmány Péter Catholic University, 1083 Budapest, Hungary; nagy.zoltan@itk.ppke.hu (Z.N.); szolgay.peter@itk.ppke.hu (P.S.); 3 Institut de Neurosciences Cognitives et Intégratives d’Aquitaine, University of Bordeaux, CEDEX, 33076 Bordeaux, France; aymar.derugy@u-bordeaux.fr

**Keywords:** FPGA, convolutional neural network, computer vision, image processing

## Abstract

The present paper proposes an implementation of a hybrid hardware–software system for the visual servoing of prosthetic arms. We focus on the most critical vision analysis part of the system. The prosthetic system comprises a glass-worn eye tracker and a video camera, and the task is to recognize the object to grasp. The lightweight architecture for gaze-driven object recognition has to be implemented as a wearable device with low power consumption (less than 5.6 W). The algorithmic chain comprises gaze fixations estimation and filtering, generation of candidates, and recognition, with two backbone convolutional neural networks (CNN). The time-consuming parts of the system, such as SIFT (Scale Invariant Feature Transform) detector and the backbone CNN feature extractor, are implemented in FPGA, and a new reduction layer is introduced in the object-recognition CNN to reduce the computational burden. The proposed implementation is compatible with the real-time control of the prosthetic arm.

## 1. Introduction and State-of-the Art

One of the problems assistive robotics addresses is the production of upper limb prostheses for amputees. Despite great progress in upper limb bionic prostheses, allowing for object-of-interest reaching and grasping, the key remaining issues relate to their control by the operator. To overcome the limitations of traditional control solely based on the electromyographic (EMG) activity of the remaining muscles, promising alternatives consider hybrid systems combining noninvasive motion capture and vision control [1,2]. They include camera vision modules that allow for recognition of the subject’s intention to grasp an object and assist visual control of prosthetic arms for object reaching and grasping [3].

The computer vision algorithms which are implemented in these systems comprise the latest object recognition approaches, such as deep neural network (DNN) classifiers and regressors [4]. In our previous work [5] we proposed an FPGA-implemented SIFT detector for matching of views in a multi-camera visual prosthesis servoing system. Despite the fact that the visual servoing of robotic arms has been a highly researched subject [6], the application to arm neuroprostheses implies supplementary constraints. The whole control device has to be lightweight and worn by the subject. Hence, it is necessary first to minimize the equipment and second to propose efficient lightweight solutions for visual scene analysis by the camera worn by the subject.

Real-time performance is also a mandatory requirement for our target application [2,7]. As the fastest visuomotor response to a perturbation takes about 90 ms [8], and feedback delays of 100 ms or more are known to deteriorate the performance of online feedback control [9], computation time should remain as low as possible, and below 100 ms.

In this work, we propose a hybrid hardware/software (HW/SW) architecture for the analysis of a visual scene for the visual servoing of a neuroprosthetic arm using a glass-worn camera. The visual task here is to recognize the object the subject intends to grasp and localize it in the egocentric visual scene.

### 1.1. State-of-the-Art Hybrid Solutions in Robotic Vision

As the core block for object recognition in our system is a convolutional neural network (CNN), we further present a brief state-of-the-art review of lightweight CNNs for object detection.

### 1.2. State-of-the-Art lightweight CNNs for Object Detection

In recent years, in the field of computer vision, the most popular algorithms for object detection are deep convolutional neural networks, such as faster regions with CNN (Fast R-CNN) [10], you only look once (YOLO) [11], and single shot detector (SSD) [12]. These detectors are based on deep residual networks (Resnet) [13], very deep convolutional networks (VGGnet) [14], Alexnet [15], MobileNet [16], and GoogleNet [17].

**Figure 1:**
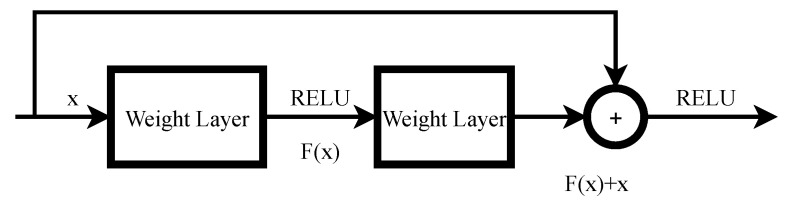
Example of the residual block in the Resnet.

Resnet [13] was proposed by He et al. and uses residual blocks, which are illustrated in Figure 1.

Denoting the desired underlying mapping as H(x).

F(x): = H(x)−x

where we let the stacked, nonlinear layer fit another mapping of F(x). The original mapping is recast into F(x)+x. It is easier to optimize the residual mapping than to optimize the original mapping. F(x)+x can be realized by feedforward neural networks with shortcut connections, as illustrated in Figure 1. Shortcut connections can skip one or more layers. In Resnet [13], the shortcut connections’ outputs are simply added to the outputs of the stacked layer.

The computational cost of the Resnet [13] is high which makes real-time implementation difficult. However, there are methods that can accelerate the computational speed.

VGGNet [14] is a simple deep convolutional neural network, where deep refers to the number of layers. The VGG-16 consists of 13 convolutional layers and 3 fully connected layers. The convolutional layers are simple because they use only 3 × 3 filters and pooling layers. This architecture has become popular in image classification problems.

Faster R-CNN [10] was proposed by Ren et al. This architecture has gained popularity among object detection algorithms. Faster R-CNN [10] is composed of the following four parts:

- feature extraction module, this can be a VGGnet [14], Mobilnet [16], or Resnet [13];
- region proposal module to generate the bounding boxes around the object;
- classification layer to detect the class of the object—for example, cat, dog, etc.;
- regression layer to make the prediction more precise.

The computational speed of the network depends on the feature extraction module and the size of the region proposal module.

Both SSD [12] and YOLO [11] are single-stage detectors. They are significantly faster than two-stage detectors (region-based methods), such as Faster R-CNN [10]. However, in cases when the objects have not so much variability, neither interclass nor intraclass Faster R-CNN [10] is a well-suited network. In our problem, we are interested in naturally cluttered home environments, where the subject intends to grasp an object, such as in kitchens. The vision analysis system we propose has to be designed to recognise objects to grasp in the video, similar to the grasping-in-the-wild (GITW) dataset [18]. This dataset was recorded in natural environments by several healthy volunteers and we made it publicly available on the CNRS NAKALA platform. The objects here, seen from the glass-mounted camera, are quite small. Their surface merely represents 10% of the whole video frame. Hence, Faster R-CNN [10] is a better choice than the SSD [12] and YOLO [11]. This is due to the fact that Faster R-CNN achieves higher mean average precision (mAP) than them, as reported by Huang et al. [19] for small objects.

The original Faster R-CNN [10] uses VGGnet [14] as a feature extractor. However, the mAP is higher when Resnet [13] is used as a backbone [20]. When the object is small, the mAP of the backbone with Resnet [13] is higher than the backbone with MobileNet [16], as reported in [19].

There are several possible ways to accelerate an algorithm [21]. In our case, FPGA was chosen in the interest of developing a lightweight and portable device [22].

Neural network inference can be very efficiently accelerated on field-programmable gate arrays (FPGA). The most important frameworks and development environments are Vitis AI [23], Apache TVM Versatile Tensor Accelerator (VTA) [24], Brevitas [25], and FINN [26].

Due to the large computing and memory bandwidth requirements, deep learning neural networks are trained on high-performance workstations, computing clusters, or GPUs using floating-point numbers. The memory access pattern of the inference step of a trained network is different, offering more data reuse and requiring smaller memory bandwidth. It makes FPGAs a versatile platform for acceleration. Computing with floating-point numbers is a resource-intensive process for FPGA in terms of digital signal processing (DSP) slices and logic resource usage. Memory bandwidth, required to load 32 bit floating-point state values and weights, can be still high compared with the capabilities of low-power FPGA devices. Additionally, a significant amount of memory is required for buffering state values and partial results in the on-chip memory of the FPGA. One possible solution would consist of using the industry standard bfloat, 16-bit, floating-point representation, which can improve the inference speed of an FPGA. Observations show [26] that the value of weights, state values, and partial results during the computation usually fall in a relatively small range and the 8-bit exponent range of the bfloat type is practically never used. If the range of the values during the computation is known in advance, then fixed-point numbers can be used. One of the major application areas of FPGAs is signal processing; therefore, the DSP slices are designed for fast, fixed-point multiply–accumulate (MAC) or multiply–add (MADD) operations, which can be utilized during neural network inference.

Converting a neural network model trained with floating-point numbers to a fixed-point FPGA-based implementation usually requires an additional step called quantization. Here, a small training set is used to determine the fixed-point weights and optimize the position of the radix point in each stage of the computation. The common bit width for quantization is 16 or 8 bits, where the accuracy of the network is slightly reduced. In some cases, even a binary representation is possible [26], eliminating all multiplications from the computation, which makes FPGA implementation very efficient while the accuracy is decreased slightly.

For latency-sensitive applications, this fixed-point model can be implemented on a streaming architecture, such as FINN [26], where layers of the network are connected directly on the FPGA. Using this structure, loading and storing state values can be avoided. In an ideal case, when the number of weights is small enough, they can be stored in the on-chip memories, further reducing the memory bandwidth requirements of the system. This also results in lower dissipated power due to the high energy requirement of off-chip data movement. Another approach used in Vitis AI [23] and Apache TVM VTA [24] is to divide the computation into a series of matrix–matrix multiplications and create a customized ISA (instruction set architecture) to execute these operations efficiently. The resulting system might have higher memory bandwidth requirements and longer latency, but can be easily reprogrammed to infer a different network during different steps of an image processing application.

Apache TVM VTA [24] is an open, generic, and customizable deep learning accelerator with a complete TVM-based compiler stack. It is an end-to-end hardware–software deep learning system stack that combines TVM and VTA. It contains the hardware design drivers, a just-in-time (JIT) runtime, and an optimizing compiler stack based on TVM.

The main advantages of the quantization are reduced complexity of the circuit, efficient use of dedicated hardware resources, reduced on-chip memory requirements, reduced off-chip memory bandwidth, and smaller power dissipation. Thus, for a lightweight body-worn device, Vitis AI [23] is a good choice, because it can accelerate the network with minimal accuracy loss.

The remainder of the paper is organized as follows. In Section 2, we present the system overview for object detection in egocentric camera view, previously developed in [4], which we further adapt. In Section 3, we propose a hybridization of the solution for the FPGA–CPU board to be incorporated into a body-worn device for prosthetic control. In Section 4, we present our results, measuring the execution time, while comparing it on different platforms. Section 5 concludes our work and outlines its perspectives.

## 2. System Overview for Object Detection

In this section, we present a system overview for object detection in egocentric video, explain each module, and propose our adaptation of a gaze-driven CNN for object recognition to meet the real-time constraints of our hybrid solution.

### 2.1. System Overview

**Figure 2:**
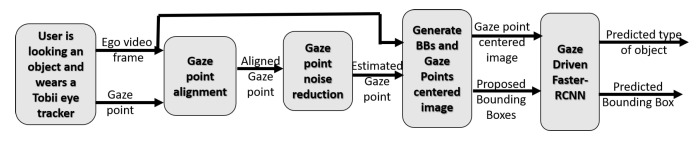
The prosthetic arm visually guided system.

The vision analysis part, which is the most critical in the whole chain of prosthesis servoing, is presented in Figure 2. The underlying hypothesis for the functioning of vision-guided neuroprostheses is that the upper limb amputee wearing the neuroprosthesis is first looking at the object they wish to grasp. The subject is wearing a Tobii glasses device, which acquires an ego-visual scene and records gaze fixations of the subject in their coordinate system—see the left-most block in Figure 2. The recorded gaze fixations allow for roughly localizing the object of interest in video frames. Nevertheless, visual saccades to the distractors in a visual scene, microsaccades, and initial scene exploration before the subject finds the object make these measurements noisy. Hence, two blocks of the system—gaze point alignment and gaze point noise reduction—serve to estimate the position of the gaze fixation on the object in the current ego-video frame. The gaze point alignment module aims to estimate and compensate for the ego-motion between the past frames and the current frame. For more details, see Section 2.2. The goal of the gaze point noise reduction module is to reduce the noise in the current frame. This noise can be a head motion, or a product of the user being distracted and looking at another object for a moment. For more details, see Section 2.3. Then, the video frame is cropped around the estimated gaze point to limit the area of the object search. Finally, different object proposal bounding boxes (BBs) at different scales are generated around the point for object localization. The gaze point-centred image and the set of BB coordinates are then submitted to the gaze-driven CNN—see the right-most block in Figure 2. The gaze-driven CNN is pre-trained on the taxonomy of objects to detect. It outputs the best score for the object class and the best-scored bounding box. When the object is localized in a video frame, the 3D position of it for prosthesis servoing can be estimated from eye tracker depth measures of gaze fixation and the coordinates of the centre of the best-scored bounding box.

The resolution of the Tobii first-person view camera is full HD (1920 × 1080 p), with a frame rate of 25 frames per second (fps). The real-time requirement for the system in our case means that each processing step of the localization of the object of interest in the glasses-mounted camera in a current video frame has to be lower than 40 ms (the video acquisition rate), and the latency of the whole system should be lower than 100 ms to leave the place for mechanical servoing of the prosthetic arm [7]. In this work, we do not consider depth estimation, which is a simple regression from eye tracker gaze fixation measures—our focus is on object detection. In the following passages, we present each system block in detail.

### 2.2. Gaze Point Alignment

The Tobii glass camera and eye tracker system output the coordinates of gaze fixations in each video frame of the first-person integrated camera.

**Figure 3:**
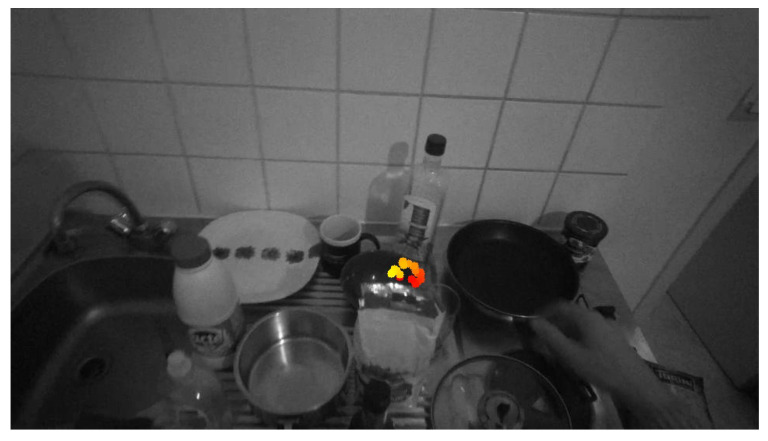
Example of bowl place 4 subject 2 gaze point alignment. The points are the gaze points.

Even if the subject is looking at the same object to grasp during the object reaching, the projected gaze points will vary between two consecutive frames because of the body and ocular movements. Furthermore, saccades provoked by distractors can deviate from the human gaze. Hence, the first step consists of the estimation of a gaze fixation in the current (reference) video frame using all the past recorded gaze fixations. It is necessary to estimate and compensate the ego-motion between the past frames and the current frame to collect all gaze points in the same reference frame. We show an illustration of such a collection in Figure 3, where the light is the gaze fixation point, and more distanced it is from the current timestamp.

Motion compensation from the past frames to the current frame is realized by a sequential homography transformation computed between consecutive frames.

Suppose a video sequence given with *N* frames and a list of gaze points, $g_{n}=\left\{\left(g_{xn},g_{yn}\right),n=1\ldots N\right\}$. The system operates as follows: for each pair of consecutive frames, it extracts the characteristic keypoints and local features. In our case, the keypoint extractor is the scale invariant feature transform [27] (SIFT). A fast library for approximate nearest neighbours (FLANN)-based matcher [28] is used to find the good matches between the SIFT descriptors of the two frames.

The final step is to estimate the homography transformation matrix, $H_{n},n=1,\ldots ,N$, with *N*, the number of the current frame, based on the good matches. Then, the gaze fixations can be projected from all frames into the current frame by a composition of homographies $H_{n}$. In this projection, we use a sliding window of duration, Δt=10, frames which correspond with 400 ms time interval, with the scene apprehension time by the subjects in our experiments. Therefore, for the current frame, *N*, the collected gaze points are ${\hat{g}}_{N,n},n=N-\Delta t,\ldots ,N$.

### 2.3. Noise Reduction

The goal of this module is to reduce the noise of the gaze fixations projected into the current frame.

**Figure 4:**
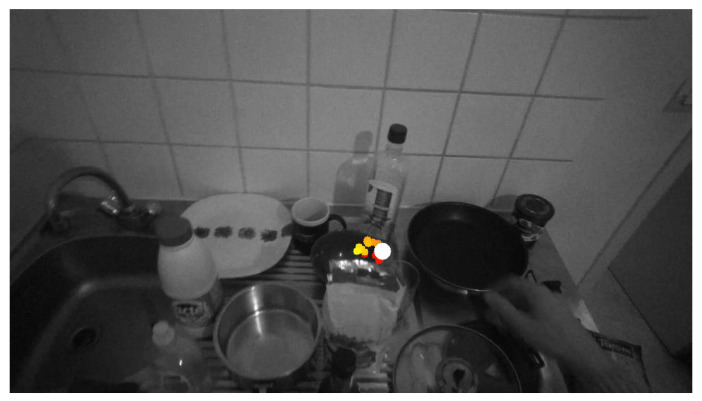
Example of bowl place 4 subject 2 KDE gaze point estimation. The points are the gaze points and the white point is the estimated gaze point.

The list of the aligned gaze fixations, ${\hat{g}}_{N,n},n=N-\Delta t,\ldots ,N$, is the input of the kernel density estimator (KDE) with Gaussian kernel [29], which predicts the most probable location of the gaze fixation in the current frame. The KDE estimates the values as described in the following equation:

$$\rho_{K}\left(y\right)=\sum_{i=1}^{L_{N}}K\left(y-{\hat{g}}_{N,n,i};h\right)$$

where a kernel, K(x,h), is a positive function that is controlled by the bandwidth parameter, *h*. In our case, the bandwidth, *h*, parameter of the Gaussian kernel was set to 1, as default. $L_{N}$ is the number of gaze points projected in the current frame *N* The maximum of the estimated density surface is considered as a predictor of the gaze fixation point in the current frame. The search for the maximum is realized inside a bounding box which encompasses all projected gaze fixations ${\hat{g}}_{N,n},n=N-\Delta t,\ldots ,N$, using full search method with pixel accuracy. An example of an estimated gaze point in a frame is presented in Figure 4, see the bright disk of the largest diameter.

### 2.4. Gaze-Driven Object Recognition CNN

This module recognizes the object location and type (e.g., bowl, pan, etc.) in a first-person video frame. A limited number of bounding boxes of different scales is generated around the estimated gaze fixation point to localize the object. The module’s input is thus the estimated gaze fixation point ${\hat{g}}_{n}$, the cropped frame around the estimated gaze fixation, and the possible bounding boxes of the object generated around ${\hat{g}}_{n}$—see the second block in Figure 2.

In the current work, 9 bounding boxes (BB) have been generated with different scale and shape factors. The size of a cropped frame is 300 × 300 px [4]. For the size of BB, we have considered the width and the height between 67 and 223, in accordance with the frame resolution and the typical object sizes in egocentric visual scenes.

Recognition of the object is carried out by a CNN classifier applied to each of the generated bounding boxes. The BB with the maximum score is thus considered as the object location.

**Figure 5:**
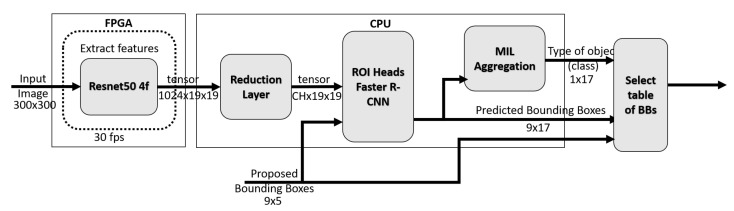
Gaze-driven, object-recognition CNN, where CH is the number of output channel of the reduction layer.

Figure 5 shows the structure of the gaze-driven CNN. The backbone is a Resnet50 in the first four layers, see the left-most block in Figure 5. These layers serve as feature extractors from the input image. The input of the backbone is a cropped video frame of size 300 px × 300 px × 3. The output is a 1024 × 19 × 19 feature tensor.

Not all feature channels are equally important for object classification when using the backbone. To select the most important ones, and to reduce the computational burden of the remaining part of the network, we introduce a reduction layer (RL). It reduces the number of channels in the input tensor to a given channel number CH (in our case, CH can be: 32, 64, 96, 128, 256, 512, 1024).

The input of RL is the backbone output tensor of dimension 1024 × 19 × 19. The RL applies a 2D convolution [30] over an input signal composed of several input planes. Assume that the input is (*N*, *C*${}_{in}$, *H*, *W*) and the output is (*N*, *C*${}_{out}$, *H*${}_{out}$, *W*${}_{out}$), then the RL can be precisely described as follows:

(1) $$out\left(M_{i},C_{{out}_{j}}\right)=bias\left(C_{{out}_{j}}\right)+\sum_{k=0}^{C_{in}-1}weight\left(C_{{out}_{j}},k\right)★input\left(M_{i},k\right)$$

where ★ is the valid 2D cross-correlation operator, *M* is the batch size, *C* denotes the number of channels, *H* is the height of input planes in pixels, and *W* is the width in pixels.

**Figure 6:**
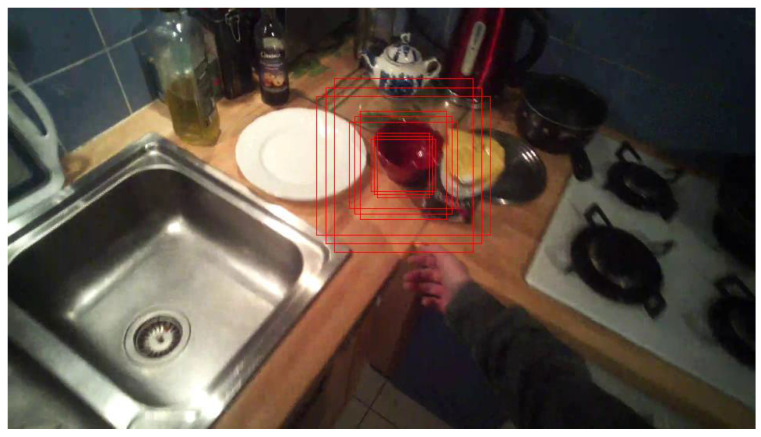
Example of bowl place 1 subject 1 generated bounding box. The bounding boxes generated around the red bowl.

Bounding boxes generated around the estimated gaze fixation point, and feature tensor with the reduced number of channels (CH× 19 × 19) are the inputs of the Faster R-CNN module [31] (ROI Heads). The module predicts the object type and location as a 17 × 9 tensor as we have 9 BBs (see Figure 6 and work with a 17-class taxonomy comprising 16 object classes and a rejection class, as in [4]. This tensor contains the probability of each bounding box for each class.

(2) $$output_{ROIheads}=\left[\begin{array}{ccccc} P_{11} & P_{12} & P_{13} & \ldots & P_{1B}\\ P_{21} & P_{22} & P_{23} & \ldots & P_{2B}\\ \; & \; & \ldots & \; & \\ P_{C1} & P_{C2} & P_{C3} & \ldots & P_{CB} \end{array}\right]$$

Equation (2) is the output tensor of the ROI heads (Faster R-CNN [31]), where Ci are the categories and *B* are the bounding boxes.

The class scores of bounding boxes are aggregated, as in [4], by multiple instance learning [32] (MIL). The input of the MIL aggregation is the output tensor of the Faster R-CNN [31]. The module predicts the class of the frame. The frame-level score ($\hat{y}\left(f,c\right)$) is calculated as shown in Equation (3).

(3) $$\hat{y}\left(f,c\right)=\frac{1}{\gamma }log\left(\sum_{b=1}^{BB_{f}}e^{\gamma y(b,c)}\right)$$

Here, *f* is the frame, *c* is the class, *b* is the bounding box, and y(b,c) is the score of the bounding box. γ is an open parameter.

MIL aggregation will produce the vector of the frame-level scores for the object categories. This vector can be finally transformed into the vector of object probabilities using a simple softmax operator: p(f, c) = softmax($\hat{y}$(f, c)).

## 3. System Hybridization

To propose a hybridization of the system, compatible with real-time performance, we have conducted thorough time measurements on different CPUs and processors to identify the most time-critical modules. The bottleneck is the Scale Invariant Feature Transform (SIFT) detector, which is required in our system for geometric alignment of gaze points—see Figure 2. The main steps of the SIFT are the following: scale-space extrema detection, keypoint localization, orientation assignment, and descriptor generation. For hardware acceleration, we have chosen Xilinx UltraScale ZCU102 [33] FPGA as it supports the parallel execution, and the energy consumption is very low. In our previous work [5], we proposed an SIFT detector on FPGA. It comprises a non-maximum suppression method to filter the keypoints which are too close, instead of the Taylor expansion in the keypoint localization step. We use this implementation in the present work.

The other complex module is the CNN for object recognition. Nevertheless, CNN is pre-trained offline for a given set of object categories. The spatial regularity of the CNN inference makes it ideal for FPGA implementation, and hundreds of papers have been published in this area in recent years. The proposed solutions can be divided into two classes: streaming architectures and parametrizable blocks.

The structure of the streaming architectures closely follows the data flow of the given network by connecting templated processing blocks in a pipeline. Input and output of the blocks are data streams (FIFO interfaces) and each operation in the network—e.g., convolution, pooling, nonlinear response, etc.—has a dedicated block for FPGA implementation [34].

The usual template parameters in the case of a convolution block are the number of input and output layers and the size of the convolution window. The input image is fed into the system in a row-wise order, which makes it possible to connect the network directly to a camera input. The latency of the resulting system is low because the convolution blocks can start processing as soon as the first rows required for the computation are available.

The main drawback of the streaming architecture is that all the weights for the computation must be stored on-chip, which is not possible for large networks. In addition, the computation load of the layers is very different. Therefore, different design optimization strategies must be used for each layer, which makes the design process complicated.

Another approach is to use a compiler to break down the entire CNN computation into a series of tensor operations and create parametrizable hardware blocks to efficiently execute them [24,35]. The fundamental building block of these architectures is a matrix–matrix multiplication block, which is usually extended by an additional functional unit to efficiently carry out other operations, such as max pooling and nonlinear transformation. The matrix–matrix multiplication is usually carried out by a systolic array of multiply–accumulate (MAC) units. A critical part of the system is the compiler, which is also responsible for the optimal scheduling of the tensor operations. The input image, network weights, and partial results are stored in off-chip memory, so the network size is not limited by the size of the FPGA device. On the other hand, the latency of the CNN computation is higher in this case because the entire image frame must be captured and stored in the memory before processing is started. Performance of the system might be also limited by the available off-chip memory bandwidth.

Taking into account the real-time constraints and also dissipation power, we implement a hybrid solution both for the preliminary processing steps before feeding gaze-driven CNN and the CNN as well. Referring to Figure 2, the hybridization of the preliminary steps is given in Table 1.

As for the gaze-driven CNN implementation, accordingly with the time measures for real-time compatibility and simplification of R-CNN input by channel number reduction we proposed—see Section 2.4—only the ResNet backbone is implemented on FPGA; as depicted in Figure 5. The details of all modules from the input of CNN to the final aggregation of decisions by MIL are given in Table 2 below.

The reference software implementation of the system was executed on a four-core Intel i5 7300HQ [36] laptop CPU running at 2.5 GHz. This software system is also compiled for the four-core ARM Cortex A53 [37] processor system (PS) of the Xilinx Zynq UltraScale+ XCZU9EG device on the ZCU102 development board. Based on these measurements, the system was partitioned between the PS and the programmable logic (PL) parts of the device. Specialized accelerator circuits were designed for the modules of the proposed system, which cannot be executed fast enough on the ARM Cortex A53 processors. A traditional register-transfer-level (RTL)-based design of a digital circuit is time consuming; therefore, the Xilinx Vitis HLS system was used to create the FPGA-based circuits from a high-level C/C++ description.

We give our measures justifying these choices and the overall results in the next section.

## 4. Results

In this section, we discuss the measured computing time of the different steps of the proposed algorithm.

### 4.1. Dataset

The GITW [18] dataset contains egocentric videos recorded by a camera on the eye tracker glasses. It includes the gaze points of where the person was looking at each moment. The videos were recorded in the wild, in real kitchens, by different subjects, and every video was recorded by a subject who grasped a kitchen object.

The acquisition device used was Tobii Glasses 2 (eye tracker) with an egocentric scene camera. The Tobii Glasses video resolution is HD (1280 pixels × 720 pixels), and the video frame rate is 25 fps. There are 16 different kitchen objects in the videos: bowl, plate, wash liquid, vinegar bottle, milk bottle, oil bottle, glass, lid, saucepan, frying pan, and mug. Different subjects recorded the dataset in five different kitchens. The videos were short, around 10 s long. The GITW [18] dataset contains 404 videos overall. The dataset is freely available for research.

We carried out the time measurements on a subset of the GITW dataset, containing fifteen videos of “grasping a bowl” actions, recorded by four different subjects. The kitchen environments are of different complexity, from a scene with just a few objects, such as the BowlPlace1 videos, to a highly cluttered scene, such as BowlPlace4. The class bowl object had a strong inner variance: different colours, the material of the bowl object, and even a transparent one. The lighting conditions and the visibility are different. Moreover, sometimes, we obtained strong blurring effects due to the camera motion, which was worn on the person’s body.

### 4.2. Geometric Alignment Measurements

For the completeness of time measures of the whole system, we present here the result of our previous work [22]. The time measures of the geometric alignment module are given in Table 3. The OpenCV [38] library 4.5.5 version was used during this experiment. The geometric alignment consists of an SIFT [27] keypoint extractor, an FLANN matcher [28], and a homography estimator. In the first part of Table 3, we give measures on embedded mobile ZCUs. The left-most column of Table 3 contains the name of the video file. The SIFT points have been detected in the mask, centred on the estimated gaze fixation point in each frame. The radius of the mask was chosen to encompass approximately 100 points. The second column contains the mean mask radius with standard deviation. For the geometric alignment by homography, we detected keypoints in two video frames: the current and previous reference frames. In the next columns, we give time figures on ARM A53 processors for keypoint (KP) computation on one frame, the matching time, and homography computation time.

In Table 3, the second column contains the number of detected SIFT points with the corresponding mask radius. We also present it as the mean and standard deviation on the whole video. The time figures are given for general purpose Intel processors.

The matcher, the homography estimator, and the gaze projection on ZCU102 are fast enough for real-time processing, as illustrated in Table 3. The worst-case scenario was 0.024 s, for the FLANN matcher [28], which means that the frame rate does not exceed 40 fps. This speed is enough for controlling a robotic arm.

However, the SIFT keypoint extractor was slower than the required processing time. While the worst-case scenario on the Intel i5 7300HQ CPU took 0.072 s, which is around 13.81 fps, on the ARM A53, it took 0.866 s, which is around 1.15 fps. For real-time processing, a rate of least 10 fps is required.

### 4.3. Kernel Density Estimation

Table 4 illustrates a comparison of the estimated time of KDE computation between the Intel i5-7300HQ and the Xilinx ZCU102 ARM Cortex A53. The second column contains the available number of gaze points during a frame gaze point estimation. The Intel i5-7300HQ [36] computes the KDE at 80 fps on average, and the ARM A53 [37] computes the KDE at 7.9 fps on average. In some critical cases, when the scattering of the subject’s gaze fixations is too strong, then the computation time is higher than in real-time, and is 3.9 s per frame, see the “Lid” sequence. Evidently, in such a case of highly cluttered scenes and problems of ocular movements, our system shows its limits.

The problem is caused by outlier gaze fixation points, which fall far away from the majority, increasing the KDE search area. The solution might be to use a simple clustering algorithm to find the outlier gaze fixation points and discard them. Since only the last 10 gaze fixation points are used, we think this clustering can be carried out in a short time.

However, if the projected gaze fixations in the current frame are sufficiently close (in the radius of 10 pixels approximately, which is the “normal case”), the ARM A53 [37] can compute the KDE in real-time.

### 4.4. Bounding Box Generation Time Measurements

The bounding box generation is fast on the Intel i5 7300HQ CPU. On average, 1 frame is processed in 0.42351 ± 0.01991 milliseconds, which is more than 2500 fps. The embedded ARM A53 processor is also fast enough to generate bounding boxes in real-time. The average computation time was 2.659 ± 0.027 milliseconds, which is more than 376 fps.

### 4.5. Gaze-Driven Object-Recognition CNN Time Measurements

Here, all measurements were taken by PyTorch. 1.6. [39].

The measurements in Table 5 show that the most time-consuming part of the CNN is the Resnet50 backbone. In every case, the backbone can process a frame in 0.09 s on Intel i5 7300 CPU, which is equal to 11 fps. On the ARM A53 processor, see Table 6, this time, presented in the second column, is even higher. It is about 1.8 s, thus giving 0.5 fps. This is below the required computational speed. Higher channel number causes larger computational complexity in the reduction layer and the Region of Interest (ROI) heads, as shown in Table 5 and Table 6. Nevertheless, with a reasonable number of channels after the reduction, not exceeding 128, these blocks run in real-time, with 82 fps for channel reduction and 25 fps for ROI heads.

The slowest part of the system was, thus, the backbone; therefore, it was implemented in FPGA. The accelerated Resnet50 CNN on ZCU102 can process an image in 0.02686 s, which is 37.23 fps. This is high enough for real-time processing.

The measurements in Table 6 show the results of the ARM A53 CPU.

### 4.6. Gaze-Driven Faster RCNN Accuracy

**Figure 7:**
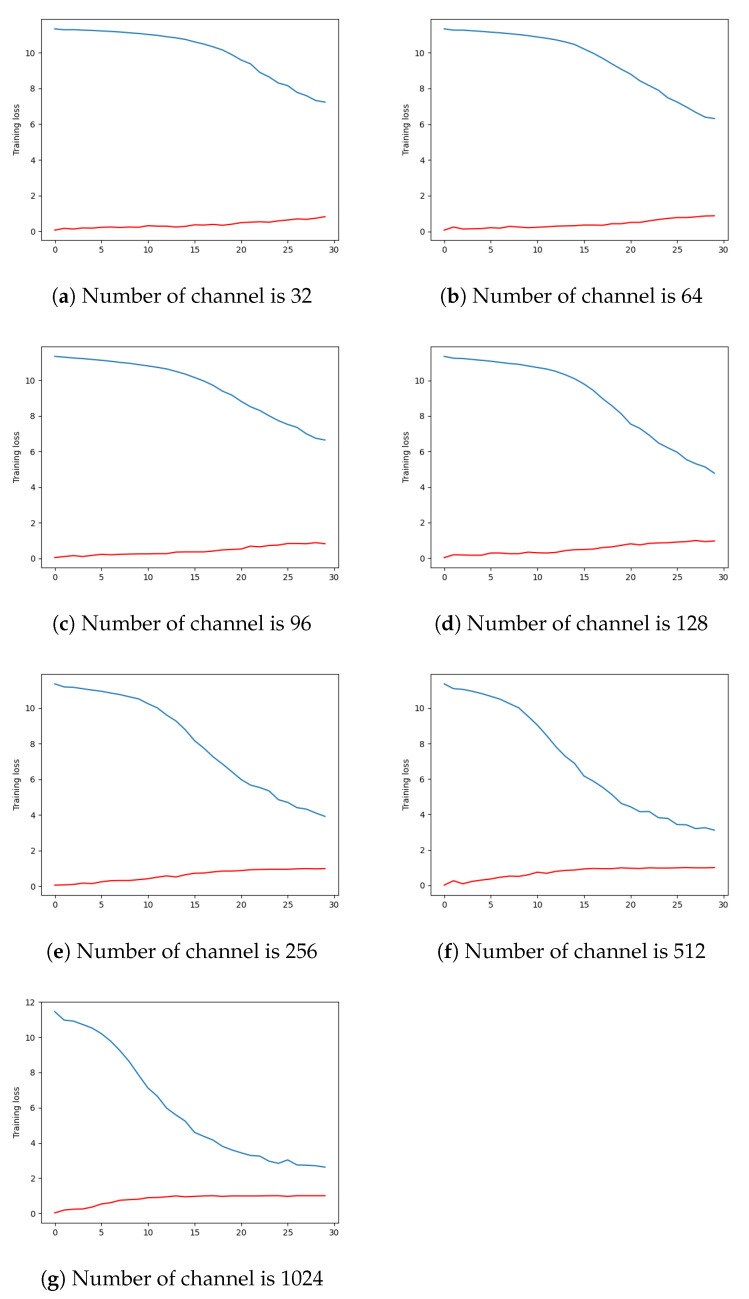
Training accuracy (in blue) and loss (in red) during 30 epochs. The top left is when the reduction layer number of channels is 32, and, next to it, 64. The vertical axis is the training loss or the accuracy, depending on the line. The horizontal axis is the epochs number.

As Table 7 and Figure 7 show, the current architecture can perform sufficiently well on our real-world data. Reducing the number of channels to 128 does not impoverish the classification accuracy too much, compared with the initial 1024 feature channels of the backbone, as we can see from Table 7. The average accuracy and loss are computed per class of objects.

Table 8 shows a comparison between different object recognition methods from the state-of-the-art methods and our method. The state-of-the-art methods, such as lightweight YOLO V3 [20] and SSD Mobilnet V2 [12], are trained on the COCO and VOC datasets. We have a specific and very cluttered kitchen environment. For this reason, we do not think that these object detectors are suitable in our case. From the computational time point of view [40], implemented on the same architecture, they are a bit faster: 13.2 fps object recognition for YOLO V3 [20] and 78.8 fps for SSD Mobilnet V2 [12]. In our work, we take profit from the availability of gaze fixations in real-time, which can drive object localization. However, the actual implementation of KDE on CPU makes the system slower. We have 12.64 fps for object recognition and its localization. The bottleneck is the KDE estimation, which we are now improving. Nevertheless, our actual computation times are compatible with real-time prosthesis control.

### 4.7. Time Measurement of the Whole System

Table 9 illustrates the average computational time of the system in milliseconds. The first row contains the module name, and the second row contains the Intel i5 7300HQ [36] CPU results. In the third row, the ARM A53 [37]-embedded CPU results are given. The fourth row contains the hybrid (ZCU102 [33] and the ARM A53 [37]) results.

The total computation time is 182.782 ms in the Intel i5 7300HQ, which is 5.471 fps. The ARM 53 [37]-embedded CPU is the slowest because it is needed 2868.066 ms per frame, which is 0.349 fps. The hybrid embedded solution is computed in a frame of 236.507 ms, which is 4.228 fps. The hybrid embedded solution is equally as fast as the Intel i5 7300HQ [36], and the power consumption of the hybrid embedded solution is 5.6 W, which is less than the Intel i5 7300HQ [36] CPU 45 W.

The measurements show that the current experimental setup with the whole chain of modules is not yet suitable for real-time processing. However, with pipelining the modules, with some delays, the real-time processing speed is achievable.

## 5. Conclusions and Perspectives

In this paper, we have proposed a hybrid implementation of a visual analysis part for visual servoing of a prosthetic arm. The system was partitioned between the FPGA fabric and the ARM Cortex A53 processors of the Xilinx ZCU102 development board, based on the computing performance measurements of the building blocks. As a reference, the computing time of each image processing step was also measured on a laptop microprocessor and its power dissipation was estimated.

The measurements show that the gaze point alignment steps are fast enough on the ARM Cortex A53 [37]-embedded CPU, except the SIFT [27] point extraction step. Therefore the SIFT [27] detection module is implemented on the programmable logic part of the Xilinx ZCU102 [33] FPGA board.

In some cases, we find that the variance of the computing time of the KDE in our current setup is very high and slows down processing. In these scenes, most of the gaze points are located over the object to grasp, except one or two, which scattered around the image due to the saccadic movement of the eye. To overcome this problem, we plan to apply an outlier filtering by clustering before KDE computation.

The gaze-driven CNN is built on 4 different modules: Resnet50 [13], reduction layer, Faster R-CNN [10], and multiple instance learning (MIL) aggregation. Resnet50 [13] was accelerated on FPGA because the measured computational speed on the ARM Cortex A53 processor was only 0.55 fps, which was improved to 37.23 fps. The Faster R-CNN is also slow, providing only 3.5 fps when the number of input channels is 1024. We thus proposed a new reduction layer between the Resnet50 [13] and the Faster R-CNN [10] to reduce the number of input channels for the latter block. The frame rate can be increased to 25 fps when the number of input channels for the Faster R-CNN is reduced to 128 by the reduction layer. The experiments show that the accuracy using only 128 channels is still high enough for the bounding box computation.

The experimental setup, with the whole chain of modules is not suitable for real-time processing (236.507 ms on average, or approximately 4 fps). However, this computing time can be improved by pipelining the system and processing different frames at each stage, because each block can finish processing an image within 40 ms. The drawback of pipelining is increased latency. The latency of our current system is around 250 ms, which is higher than the latency allowed by the control of the robotic arm (∼100 ms) and is mainly caused by the KDE block. In the future, the KDE search algorithm will be optimized.

The power consumption and processing speed for the different architectures show that the embedded system, accelerated with FPGA, is a feasible solution for creating a wearable device.

## Figures and Tables

**Table 1:** Hybridization of preliminary steps in the pipeline, which contains two main blocks: Gaze-Point Alignment Block and Gaze-Point Noise Reduction Block and its submodules.

Module	CPU	FPGA
**Gaze-Point Alignment Block **		
SIFT Detection [5]	-	X
SIFT Matching	X	-
Homography estimation	X	-
Gaze-point projection	X	-
**Gaze-Point Noise Reduction Block**		
KDE estimation	X	-

**Table 2:** Hybridization of the gaze-driven CNN.

Module	CPU	FPGA
Resnet50	-	X
Reduction layer	X	-
Faster R-CNN	X	-
MIL aggregation	X	-

**Table 3:** Comparison between the Intel i5 7300HQ and the Xilinx ZCU102 ARM CORTEX A53.

		Xilinx ZCU102 ARM CORTEX A53			
**Video File Name**	**Mask Radius**	**SIFT KP Extractions (ms)**	**Matcher (ms)**	**Homography (ms)**	**Gaze Projection (ms)**
BowlPlace1Subject1	119 ± 25	875.504 ± 12.123	23.471 ± 5.203	2.200 ± 0.540	0.089 ± 0.004
BowlPlace1Subject2	106 ± 16	875.282 ± 9.504	20.036 ± 3.704	1.900 ± 0.398	0.088 ± 0.001
BowlPlace1Subject3	153 ± 50	873.072 ± 7.283	17.626 ± 3.276	2.539 ± 0.621	0.089 ± 0.001
BowlPlace1Subject4	120 ± 25	873.545 ± 9.062	22.244 ± 5.938	2.160 ± 0.464	0.092 ± 0.009
BowlPlace4Subject1	158 ± 55	855.947 ± 6.583	16.011 ± 3.053	2.883 ± 1.188	0.088 ± 0.001
BowlPlace4Subject2	117 ± 24	861.933 ± 5.821	16.276 ± 2.623	1.997 ± 0.449	0.089 ± 0.004
BowlPlace4Subject3	108 ± 19	867.649 ± 8.894	15.679 ± 4.620	2.136 ± 0.350	0.089 ± 0.005
BowlPlace4Subject4	147 ± 49	857.271 ± 9.468	16.762 ± 4.186	2.240 ± 0.516	0.088 ± 0.001
BowlPlace5Subject1	120 ± 33	861.481 ± 8.012	17.875 ± 2.176	2.018 ± 0.505	0.088 ± 0.001
BowlPlace5Subject2	133 ± 42	858.547 ± 6.232	17.944 ± 3.024	2.354 ± 0.880	0.088 ± 0.001
BowlPlace5Subject3	126 ± 33	859.774 ± 6.384	15.742 ± 2.836	2.007 ± 0.524	0.087 ± 0.001
BowlPlace6Subject1	120 ± 25	867.344 ± 10.950	19.026 ± 3.862	1.965 ± 0.306	0.088 ± 0.001
BowlPlace6Subject2	129 ± 35	862.750 ± 9.731	19.737 ± 4.973	3.681 ± 3.456	0.090 ± 0.008
BowlPlace6Subject3	127 ± 31	864.429 ± 6.931	17.555 ± 3.806	2.588 ± 0.823	0.087 ± 0.001
BowlPlace6Subject4	112 ± 22	867.962 ± 9.579	17.368 ± 4.725	2.710 ± 0.649	0.089 ± 0.004
		**Intel i5 7300HQ**			
**Video File Name**	**Number of Extracted KP**	**SIFT KP Extractions (ms)**	**Matcher (ms)**	**Homography (ms)**	**Gaze Projection (ms)**
BowlPlace1Subject1	151 ± 67	74.205 ± 5.611	3.891 ± 0.853	0.259 ± 0.051	0.015 ± $10^{-4}$
BowlPlace1Subject2	156 ± 37	75.062 ± 5.640	3.304 ± 0.579	0.228 ± 0.040	0.014 ± $10^{-4}$
BowlPlace1Subject3	86 ± 50	72.217 ± 2.572	3.011 ± 0.476	0.282 ± 0.055	0.014 ± $10^{-4}$
BowlPlace1Subject4	138 ± 69	72.979 ± 2.853	3.717 ± 0.940	0.252 ± 0.044	0.015 ± 0.002
BowlPlace4Subject1	94 ± 50	70.068 ± 2.405	2.747 ± 0.565	0.313 ± 0.113	0.014 ± $10^{-4}$
BowlPlace4Subject2	121 ± 28	72.280 ± 3.538	2.778 ± 0.407	0.233 ± 0.040	0.015 ± $10^{-4}$
BowlPlace4Subject3	126 ± 39	73.402 ± 3.406	2.678 ± 0.728	0.256 ± 0.047	0.014 ± $10^{-4}$
BowlPlace4Subject4	95 ± 50	70.394 ± 2.349	2.872 ± 0.695	0.259 ± 0.051	0.014 ± $10^{-4}$
BowlPlace5Subject1	129 ± 39	71.990 ± 2.691	3.027 ± 0.369	0.244 ± 0.050	0.015 ± $10^{-4}$
BowlPlace5Subject2	120 ± 56	71.587 ± 2.526	3.077 ± 0.573	0.272 ± 0.087	0.014 ± $10^{-4}$
BowlPlace5Subject3	108 ± 36	71.359 ± 2.500	2.684 ± 0.448	0.234 ± 0.049	0.015 ± 0.001
BowlPlace6Subject1	132 ± 48	72.150 ± 2.891	3.213 ± 0.645	0.237 ± 0.031	0.015 ± $10^{-4}$
BowlPlace6Subject2	129 ± 59	71.790 ± 3.934	3.348 ± 0.823	0.390 ± 0.316	0.015 ± $10^{-4}$
BowlPlace6Subject3	114 ± 47	72.042 ± 2.883	2.976 ± 0.617	0.287 ± 0.076	0.015 ± 0.001
BowlPlace6Subject4	138 ± 44	74.585 ± 4.431	3.089 ± 0.849	0.303 ± 0.075	0.015 ± $10^{-4}$

**Table 4:** Comparison in processing time of kernel density estimation module between the Intel i5 7300HQ and the Xilinx ZCU102 ARM CORTEX A53.

		Xilinx ZCU102 ARM CORTEX A53		Intel i5 7300HQ	
**Video File Name**	**Gaze Points**	**Time (ms)**	**Max Time (ms)**	**Time (ms)**	**Max Time (ms)**
Bowl	22 ± 8	49.27 ± 82.83	307.34	4.94 ± 7.68	27.90
CanOfCocaCola	26 ± 11	75.54 ± 95.89	395.08	7.46 ± 8.80	36.70
FryingPan	24 ± 9	59.09 ± 50.06	206.76	5.86 ± 4.51	18.98
Glass	29 ± 10	148.22 ± 265.60	943.19	14.89 ± 26.23	92.21
Jam	27 ± 12	132.75 ± 319.01	1365.65	13.34 ± 31.39	134.68
Lid	29 ± 16	247.21 ± 718.32	3835.30	23.92 ± 70.97	379.64
MilkBottle	28 ± 10	114.95 ± 148.60	647.86	11.20 ± 13.99	61.92
Mug	28 ± 11	109.88 ± 218.40	1087.39	11.03 ± 21.26	106.63
OilBottle	30 ± 12	235.15 ± 477.79	2117.26	22.86 ± 46.23	205.83
Plate	32 ± 14	203.39 ± 406.91	1837.70	19.59 ± 39.46	178.97
Rice	29 ± 13	90.34 ± 95.16	372.93	8.64 ± 8.92	35.80
SaucePan	25 ± 12	139.07 ± 261.08	1286.11	13.68 ± 25.82	126.92
Sponge	24 ± 10	50.05 ± 49.79	207.89	5.10 ± 4.76	20.46
Sugar	27 ± 14	146.60 ± 271.58	1165.44	14.46 ± 26.70	117.57
VinegarBottle	28 ± 13	122.32 ± 178.37	683.56	12.23 ± 17.71	70.01
WashLiquid	28 ± 12	102.93 ± 183.02	880.47	10.42 ± 18.45	89.25

**Table 5:** Measurements of the gaze-driven, object-recognition CNN in the Intel i5 7300 CPU. The first column contains the reaming number of channels after the reduction layer. Each column shows the elapsed time during the computation in milliseconds.

Number of Channel	Backbone (ms)	Reduction Layer (ms)	ROI Heads (ms)	Aggregation (ms)
32	90.000 ± 0.250	0.336 ± $10^{-4}$	1.107 ± $10^{-4}$	0.137 ± $10^{-6}$
64	97.307 ± 1.613	0.531 ± 0.002	2.262 ± 0.004	0.138 ± $10^{-6}$
96	87.441 ± 0.508	0.557 ± 0.003	2.956 ± 0.003	0.241 ± $10^{-4}$
128	89.952 ± 2.568	0.646 ± 0.001	3.356 ± 0.001	0.142 ± $10^{-6}$
256	85.287 ± 0.375	0.908 ± $10^{-4}$	6.592 ± 0.002	0.150 ± $10^{-5}$
512	94.505 ± 2.100	2.485 ± 0.002	12.276 ± 0.002	0.159 ± $10^{-6}$
1024	95.515 ± 7.285	3.204 ± 0.007	23.718 ± 0.010	0.164 ± $10^{-6}$

**Table 6:** Measurements of the gaze-driven, object-recognition CNN in the ARM A53 CPU. The first column contains the reaming number of the channels after the reduction layer. Each column shows the elapsed time during the computation in milliseconds.

Number of Channel	Backbone (ms)	Reduction Layer (ms)	ROI Heads (ms)	Aggregation (ms)
32	1863.300 ± 11.433	6.949 ± 0.001	13.843 ± 0.002	0.643 ± 0.001
64	1768.616 ± 15.615	8.156 ± 0.001	21.859 ± 0.006	0.708 ± $10^{-4}$
96	1787.737 ± 15.903	10.178 ± 0.001	30.705 ± 0.001	0.758 ± $10^{-6}$
128	1800.327 ± 17.915	12.140 ± 0.001	39.371 ± 0.002	0.727 ± $10^{-5}$
256	1797.798 ± 16.372	22.061 ± 0.011	73.750 ± 0.002	0.714 ± $10^{-4}$
512	1733.458 ± 14.429	33.723 ± 0.001	142.231 ± 0.001	0.752 ± $10^{-6}$
1024	1761.748 ± 16.305	63.319 ± 0.001	285.121 ± 0.002	0.714 ± $10^{-6}$

**Table 7:** The results of the training and testing after 30 epochs.

Number of Channel	32	64	96	128	256	512	1024
avg loss on training set	7.235	6.318	6.642	4.778	3.920	3.115	2.623
avg acc on trainig set	0.815	0.877	0.827	0.963	0.988	1.000	1.000
avg acc on test set	0.793 ± 0.261	0.926 ± 0.120	0.853 ± 0.161	0.952 ± 0.083	1.000	1.000	1.000
avg ap on test set	0.978 ± 0.043	0.985 ± 0.030	0.964 ± 0.041	0.995 ± 0.012	1.000	1.000	1.000

**Table 8:** Comparison of different object recognition CNNs. All the measurements were taken by Vitis AI 1.4. The gaze-driven, object-recognition CNN used 128 channels in the reduction layer.

Name	Gaze-Driven, Object-Recognition CNN	SSD Mobilnet V2	YOLO V3
Dataset	GITW	COCO	VOC
Framework	Pytorch	Tensorflow	Tensorflow
Input size	300 × 300	300 × 300	416 × 416
Running device	ZCU 102 + ARM A53	ZCU 102	ZCU 102
FPS	12.64	78.8	13.2

**Table 9:** The average computational time measurement of the whole system on different hardware. The Resnet50 number of channels is 128.

	Computational Time (ms)		
**Module Name**	**Intel i5 7300HQ CPU**	**ARM A53**	**FPGA + ARM A53**
SIFT [27]	72.407 ± 3.349	865.499 ± 8.437	7.407 [5]
FLANN matcher	3.094 ± 0.638	18.223 ± 3.867	18.223 ± 3.867
Homography estimation	0.270 ± 0.075	2.359 ± 0.778	2.359 ± 0.778
Gaze point projection	0.015 ± $10^{-4}$	0.089 ± 0.003	0.089 ± 0.003
KDE estimation	12.477 ± 23.306	126.672 ± 238.900	126.672 ± 238.900
Bounding Box generation	0.424 ± 0.020	2.659 ± 0.027	2.659 ± 0.027
Resnet50 [13]	89.952 ± 2.568	1800.327 ± 17.915	26.860
Reduction Layer	0.645 ± 0.001	12.140 ± 0.001	12.140 ± 0.001
Faster R-CNN [10]	3.356 ± 0.001	39.371 ± 0.002	39.371 ± 0.002
MIL Aggregation	0.142 ± $10^{-6}$	0.727 ± $10^{-6}$	0.727 ± $10^{-6}$
Total time (ms)	182.782 ± 29.957	2868.066 ± 269.930	236.507 ± 243.578

## Data Availability

Publicly available datasets were analyzed in this study. This data can be found here: Grasping-in-the-wild (GITW) dataset at NAKALA CNRS server https://www.labri.fr/projet/AIV/graspinginthewild.php, accessed on 30 December 2021.

## Abbreviations

The following abbreviations are used in this manuscript:

BB	bounding box
Resnet	deep residual networks
CNN	convolutional neural network
SIFT	scale invariant feature transform
KDE	kernel density estimation
MIL	multiple instance learning
FPGA	field-programmable gate array
FPS	frame rate per second
FLANN	fast library for approximate nearest neighbors
Faster R-CNN	faster regions with CNN
KP	keypoints
GITW	grasping-in-the-wild dataset
DNN	deep neural network
EMG	electromyography
YOLO	you only look once
SSD	single shot detector
VGGnet	very deep convolutional networks
DSP	digital signal processing
MAC	multiply–accumulate
MADD	multiply–add
RL	reduction layer
